# Supplementary material for: Smooth Interpolating Curves with Local Control and Monotone Alternating Curvature
Source: Comput Graph Forum. 2022 Oct 6;41(5):25–38. doi: 10.1111/cgf.14600 (PMC9827861; doi:10.1111/cgf.14600)
Supplement: Supplementary file 1 — Supplement Material [file CGF-41-25-s001.zip › Local-Smooth-Interpolating-MonoCurvature/extern/clothoids/docs/api-matlab/program_listing_file_ClothoidList.m.html]

Program Listing for File ClothoidList.m — Clothoids v2.0.9

### Navigation

- index
- toc
- Clothoids »
- Program Listing for File ClothoidList.m

# Program Listing for File ClothoidList.m¶

↰ Return to documentation for file (`ClothoidList.m`)

```
classdef ClothoidList < CurveBase

  methods
    % - - - - - - - - - - - - - - - - - - - - - - - - - - - - - - - - - - -
    %> Create a new C++ class instance for the clothoid list object
    %>
    %> **Usage:**
    %>
    %> \rst
    %> .. code-block:: matlab
    %>
    %>    ref = ClothoidCurve()
    %>
    %> \endrst
    %>
    %> **On output:**
    %>
    %> - `ref`: reference handle to the object instance
    %>
    function self = ClothoidList()
      self@CurveBase( 'ClothoidListMexWrapper' );
      self.objectHandle = ClothoidListMexWrapper( 'new' );
    end
    % - - - - - - - - - - - - - - - - - - - - - - - - - - - - - - - - - - -
    function str = is_type( ~ )
      str = 'ClothoidList';
    end
    % - - - - - - - - - - - - - - - - - - - - - - - - - - - - - - - - - - -
    %> Reserve memory for `N` segments
    %>
    %> **Usage:**
    %>
    %> \rst
    %> .. code-block:: matlab
    %>
    %>    ref.reserve( N );
    %>
    %> \endrst
    %>
    function reserve( self, N )
      ClothoidListMexWrapper( 'reserve', self.objectHandle, N );
    end
    % - - - - - - - - - - - - - - - - - - - - - - - - - - - - - - - - - - -
    %>
    %> Save the clothoid list to a file
    %>
    function save( self, fname )
      ClothoidListMexWrapper( 'save', self.objectHandle, fname );
    end
    % - - - - - - - - - - - - - - - - - - - - - - - - - - - - - - - - - - -
    %>
    %> Load the clothoid list from a file (check consistency of the readed segments)
    %>
    %> **Usage:**
    %>
    %> \rst
    %> .. code-block:: matlab
    %>
    %>    ref.load( filename );
    %>    ref.load( filename, tol );
    %>
    %> \endrst
    %>
    %> `filename` : file name to be read
    %> `tol`      : tolerance used to check consistency
    %>
    function load( self, varargin ) % file, tol
      ClothoidListMexWrapper( 'load', self.objectHandle, varargin{:} );
    end
    % - - - - - - - - - - - - - - - - - - - - - - - - - - - - - - - - - - -
    %>
    %> Append a curve to the clothoid list
    %>
    %> **Usage:**
    %>
    %> \rst
    %> .. code-block:: matlab
    %>
    %>    ref.push_back( obj ); % mode 1
    %>    ref.push_back( kappa0, dkappa, L ); % mode 2
    %>    ref.push_back( x0, y0, theta0, kappa0, dkappa, L ); % mode 3
    %>
    %> \endrst
    %>
    %> **Mode 1**
    %>
    %> - `obj` : curve to be appended can be one of
    %>   - *LineSegment*
    %>   - *BiArc*
    %>   - *BiarcList*
    %>   - *CircleArc*
    %>   - *ClothoidCurve*
    %>   - *ClothoidList*
    %>   - *PolyLine*
    %>
    %> **Mode 2**
    %>
    %> - `kappa0` : initial curvature of the appended clothoid
    %> - `dkappa` : derivative of the curvature of the appended clothoid
    %> - `L`      : length of the the appended clothoid
    %>
    %> **Mode 3**
    %>
    %> - `x0`, `y0` : initial position of the appended clothoid,
    %>                the builded clothoid will be translated to
    %>                the and of the actual clothoid list
    %> - `theta0` : initial curvature of the appended clothoid
    %> - `kappa0` : initial curvature of the appended clothoid
    %> - `dkappa` : derivative of the curvature of the appended clothoid
    %> - `L`      : length of the the appended clothoid
    %>
    function push_back( self, varargin )
      if nargin == 2
        ClothoidListMexWrapper( ...
          'push_back', self.objectHandle, varargin{1}.is_type(), varargin{1}.obj_handle() ...
        );
      else
        ClothoidListMexWrapper( 'push_back', self.objectHandle, varargin{:} );
      end
    end
    % - - - - - - - - - - - - - - - - - - - - - - - - - - - - - - - - - - -
    %>
    %> Append a curve to the clothoid list
    %>
    %> **Usage:**
    %>
    %> \rst
    %> .. code-block:: matlab
    %>
    %>    ref.push_back( x1,y1,theta1 ); % mode 1
    %>    ref.push_back( x0,y0,theta0,x1,y1,theta1); % mode 2
    %>
    %> \endrst
    %>
    %> **Mode 1**
    %>
    %> Build a clothoid arc using final point and angle of the
    %> clothoid list and
    %>
    %> - `x1`, `y1` : final point
    %> - `theta1`   : final angle
    %>
    %> the builded clothoid is appended to the list
    %>
    %> **Mode 2**
    %>
    %> Build a clothoid arc using the data
    %>
    %> - `x0`, `y0` : initial point
    %> - `theta0`   : initial angle
    %> - `x1`, `y1` : final point
    %> - `theta1`   : final angle
    %>
    %> the builded clothoid is appended to the list
    %>
    function push_back_G1( self, varargin )
      ClothoidListMexWrapper( 'push_back_G1', self.objectHandle, varargin{:} );
    end
    % - - - - - - - - - - - - - - - - - - - - - - - - - - - - - - - - - - -
    %> Get the clothoid at position `k`.
    %> The biarc is returned as a clothoid object or the data
    %> defining the clothoid.
    %>
    %> \rst
    %> .. code-block:: matlab
    %>
    %>   [ x0, y0, theta0, kappa0, dkappa, L ] = ref.get(k);
    %>   C = ref.get(k);
    %> \endrst
    %>
    function varargout = get( self, k )
      [ x0, y0, theta0, k0, dk, L ] = ClothoidListMexWrapper( 'get', self.objectHandle, k );
      if nargout == 1
        varargout{1} = ClothoidCurve( x0, y0, theta0, k0, dk, L );
      elseif nargout == 6
        varargout{1} = x0;
        varargout{2} = y0;
        varargout{3} = theta0;
        varargout{4} = k0;
        varargout{5} = dk;
        varargout{6} = L;
      else
        error('expected 1 or 6 outout arguments');
      end
    end
    % - - - - - - - - - - - - - - - - - - - - - - - - - - - - - - - - - - -
    %> Append a curve to a clothoid list.
    %>
    %> \rst
    %> .. code-block:: matlab
    %>
    %>   C = LineSegment( ... );
    %>   ...
    %>   C = Biarc( ... );
    %>   ...
    %>   C = CircleArc( ... );
    %>   % ....
    %>   ref.append(C); % append biarc
    %> \endrst
    %>
    function append( self, lst )
      ClothoidListMexWrapper( 'push_back', self.objectHandle, lst.is_type(), lst.obj_handle() );
    end
    % - - - - - - - - - - - - - - - - - - - - - - - - - - - - - - - - - - -
    %>
    %> Return curvilinear coordinates, angle and curvature
    %> at node point for the clothoid list
    %>
    %> \rst
    %>
    %> .. code-block:: matlab
    %>
    %>  [ s, theta, kappa ] = ref.getSTK();
    %>
    %> \endrst
    %>
    %> - `s`     curvilinear coordinates nodes
    %> - `theta` angles at nodes
    %> - `kappa` curvature at nodes
    %>
    function [ s, theta, kappa ] = getSTK( self )
      [ s, theta, kappa ] = ClothoidListMexWrapper( 'getSTK', self.objectHandle );
    end
    % - - - - - - - - - - - - - - - - - - - - - - - - - - - - - - - - - - -
    %>
    %> Return xy-coordinates at node points for the clothoid list
    %>
    %> \rst
    %>
    %> .. code-block:: matlab
    %>
    %>  [ x, y ] = ref.getXY();
    %>
    %> \endrst
    %>
    %> - `x` x-coordinates at nodes
    %> - `y` y-coordinates at nodes
    %>
    function [ x, y ] = getXY( self )
      [ x, y ] = ClothoidListMexWrapper( 'getXY', self.objectHandle );
    end
    % - - - - - - - - - - - - - - - - - - - - - - - - - - - - - - - - - - -
    % Number of segments of the clothoid list
    %
    function N = numSegments( self )
      N = ClothoidListMexWrapper( 'numSegments', self.objectHandle );
    end
    % - - - - - - - - - - - - - - - - - - - - - - - - - - - - - - - - - - -
    function ok = build_3arcG2( self, x0, y0, theta0, kappa0, ...
                                      x1, y1, theta1, kappa1 )
      ok = ClothoidListMexWrapper( ...
        'build_3arcG2', self.objectHandle, ...
        x0, y0, theta0, kappa0, ...
        x1, y1, theta1, kappa1 ...
      );
    end
    % - - - - - - - - - - - - - - - - - - - - - - - - - - - - - - - - - - -
    function ok = build_3arcG2fixed( self, s0, x0, y0, theta0, kappa0, ...
                                           s1, x1, y1, theta1, kappa1 )
      ok = ClothoidListMexWrapper( ...
        'build_3arcG2fixed', ...
        self.objectHandle, ...
        s0, x0, y0, theta0, kappa0, ...
        s1, x1, y1, theta1, kappa1 ...
      );
    end
    % - - - - - - - - - - - - - - - - - - - - - - - - - - - - - - - - - - -
    function ok = build_2arcG2( self, x0, y0, theta0, kappa0, ...
                                      x1, y1, theta1, kappa1 )
      ok = ClothoidListMexWrapper( ...
        'build_2arcG2', self.objectHandle, ...
        x0, y0, theta0, kappa0, ...
        x1, y1, theta1, kappa1 ...
      );
    end
    % - - - - - - - - - - - - - - - - - - - - - - - - - - - - - - - - - - -
    function ok = build_CLC( self, x0, y0, theta0, kappa0, ...
                                   x1, y1, theta1, kappa1 )
      ok = ClothoidListMexWrapper( ...
        'build_CLC', self.objectHandle, ...
        x0, y0, theta0, kappa0, ...
        x1, y1, theta1, kappa1 ...
      );
    end
    % - - - - - - - - - - - - - - - - - - - - - - - - - - - - - - - - - - -
    %>
    %> Given a list of xy-coordinates at node points build a clothoid list.
    %> If third argument (`angle`) is not present angles are estimated internally.
    %>
    %> \rst
    %>
    %> .. code-block:: matlab
    %>
    %>  ref.build_G1( x, y );
    %>  ref.build_G1( x, y, theta );
    %>
    %> \endrst
    %>
    %> - `x` x-coordinates at nodes
    %> - `y` y-coordinates at nodes
    %> - `theta` angle at nodes
    %>
    function ok = build_G1( self, x, y, varargin )
      ok = ClothoidListMexWrapper( ...
        'build_G1', self.objectHandle, x, y, varargin{:} ...
      );
    end
    % - - - - - - - - - - - - - - - - - - - - - - - - - - - - - - - - - - -
    %>
    %> Given a list of curvilinear coordinated and curvatures
    %> at nodes build a G2 clothoid list.
    %> Initial position and angle must be set to determine a unique clothoid list.
    %>
    %> \rst
    %>
    %> .. code-block:: matlab
    %>
    %>  ref.build( x0, y0, theta0, s, kappa );
    %>
    %> \endrst
    %>
    %> - `x0`     initial x-coordinates
    %> - `y0`     initial y-coordinates at nodes
    %> - `theta0` initial angle
    %> - `s`      list of curvilinear coordinates
    %> - `kappa`  list of curvatures at nodes
    %>
    function ok = build( self, x0, y0, theta0, s, kappa )
      ok = ClothoidListMexWrapper( ...
        'build', self.objectHandle, x0, y0, theta0, s, kappa ...
      );
    end
    % - - - - - - - - - - - - - - - - - - - - - - - - - - - - - - - - - - -
    %>
    %> Given a list of xy-coordinates at node points build a guess of angles
    %> for the clothoid list.
    %>
    %> \rst
    %>
    %> .. code-block:: matlab
    %>
    %>  theta = ref.build_theta( x, y );
    %>
    %> \endrst
    %>
    %> - `x` x-coordinates at nodes
    %> - `y` y-coordinates at nodes
    %>
    function [theta,ok] = build_theta( self, x, y )
      [theta,ok] = ClothoidListMexWrapper( 'build_theta', self.objectHandle, x, y );
    end
    % - - - - - - - - - - - - - - - - - - - - - - - - - - - - - - - - - - -
    function ok = build_raw( self, x, y, abscissa, theta, kappa )
      ok = ClothoidListMexWrapper( ...
        'build_raw', self.objectHandle, x, y, abscissa, theta, kappa ...
      );
    end
    % - - - - - - - - - - - - - - - - - - - - - - - - - - - - - - - - - - - - -
    function make_closed( self )
      ClothoidListMexWrapper( 'make_closed', self.objectHandle );
    end
    % - - - - - - - - - - - - - - - - - - - - - - - - - - - - - - - - - - - - -
    function make_open( self )
      ClothoidListMexWrapper( 'make_open', self.objectHandle );
    end
    % - - - - - - - - - - - - - - - - - - - - - - - - - - - - - - - - - - - - -
    function ok = is_closed( self )
      ok = ClothoidListMexWrapper( 'is_closed', self.objectHandle );
    end
    % - - - - - - - - - - - - - - - - - - - - - - - - - - - - - - - - - - -
    function dtheta = deltaTheta( self )
      dtheta = ClothoidListMexWrapper( 'deltaTheta', self.objectHandle );
    end
    % - - - - - - - - - - - - - - - - - - - - - - - - - - - - - - - - - - -
    function dkappa = deltaKappa( self )
      dkappa = ClothoidListMexWrapper( 'deltaKappa', self.objectHandle );
    end
    % - - - - - - - - - - - - - - - - - - - - - - - - - - - - - - - - - - -
    function [X,Y,S,DST] = closestPointBySample( self, qx, qy, ds )
      [ X, Y, S, DST ] = ...
        ClothoidListMexWrapper( ...
          'closestPointBySample', self.objectHandle, qx, qy, ds ...
        );
    end
    % - - - - - - - - - - - - - - - - - - - - - - - - - - - - - - - - - - -
    function [DST,S] = distanceBySample( self, qx, qy, ds )
      [ DST, S ] = ...
        ClothoidListMexWrapper( ...
          'distanceBySample', self.objectHandle, qx, qy, ds ...
        );
    end
    % - - - - - - - - - - - - - - - - - - - - - - - - - - - - - - - - - - -
    function iseg = closestSegment( self, qx, qy )
      iseg = ClothoidListMexWrapper( ...
        'closestSegment', self.objectHandle, qx, qy ...
      );
    end
    % - - - - - - - - - - - - - - - - - - - - - - - - - - - - - - - - - - -
    function varargout = closestPointInRange( self, qx, qy, ibegin, iend, varargin )
      [ varargout{1:nargout} ] = ClothoidListMexWrapper( ...
        'closestPointInRange', self.objectHandle, ...
        qx, qy, ibegin, iend, varargin{:} ...
      );
    end
    % - - - - - - - - - - - - - - - - - - - - - - - - - - - - - - - - - - -
    %>
    %> Find closest point of a Clothoid list given a s-range
    %>
    %> \rst
    %>
    %> .. code-block:: matlab
    %>
    %>  [ icurve, x, y, s, t, iflag, dst ] = ...
    %>     ref.closestPointInSRange( qx, qy, s_begin, s_end, varargin );
    %>
    %>  [ icurve, x, y, s, t, iflag, dst ] = ...
    %>     ref.closestPointInSRange( qx, qy, s_begin, s_end, varargin, 'ISO' );
    %>
    %>  [ icurve, x, y, s, t, iflag, dst ] = ...
    %>     ref.closestPointInSRange( qx, qy, s_begin, s_end, varargin, 'SAE' );
    %>
    %>  res = ref.closestPointInSRange( qx, qy, s_begin, s_end, varargin );
    %>  res = ref.closestPointInSRange( qx, qy, s_begin, s_end, varargin, 'ISO' );
    %>  res = ref.closestPointInSRange( qx, qy, s_begin, s_end, varargin, 'SAE' );
    %>
    %>  %
    %>  % res is a struct with field
    %>  %
    %>  % res.icurve = number of the segment with the projected point
    %>  % res.x      = projected x
    %>  % res.y      = projected y
    %>  % res.s      = curvilinear coordinate of the projection
    %>  % res.t      = normal curvilinear coordinate ('ISO' or 'SAE' convenction)
    %>  % res.iflag  = 1 OK -1 projection failed
    %>  % res.dst    = point curve distance
    %>  %
    %>
    %> \endrst
    %>
    function varargout = closestPointInSRange( self, qx, qy, s_begin, s_end, varargin )
      [ varargout{1:nargout} ] = ClothoidListMexWrapper( ...
        'closestPointInSRange', self.objectHandle, qx, qy, s_begin, s_end, varargin{:} ...
      );
    end
    % - - - - - - - - - - - - - - - - - - - - - - - - - - - - - - - - - - -
    function idx = s_to_index( self, s )
      idx = ClothoidListMexWrapper( 's_to_index', self.objectHandle, s );
    end
    % - - - - - - - - - - - - - - - - - - - - - - - - - - - - - - - - - - -
    function export_table( self, filename )
      ClothoidListMexWrapper( 'export_table', self.objectHandle, filename );
    end
    % - - - - - - - - - - - - - - - - - - - - - - - - - - - - - - - - - - -
    function export_ruby( self, filename )
      ClothoidListMexWrapper( 'export_ruby', self.objectHandle, filename );
    end
    % - - - - - - - - - - - - - - - - - - - - - - - - - - - - - - - - - - -
    function info( self )
      ClothoidListMexWrapper( 'info', self.objectHandle );
    end
    % - - - - - - - - - - - - - - - - - - - - - - - - - - - - - - - - - - -
    function [ s, t, ipos ] = find_coord1( self, x, y, varargin )
      [ s, t, ipos ] = ClothoidListMexWrapper( ...
        'findST1', self.objectHandle, x, y, varargin{:} ...
      );
    end
    % - - - - - - - - - - - - - - - - - - - - - - - - - - - - - - - - - - -
    %> Plot the clothoid list
    %>
    %> **Usage:**
    %>
    %> \rst
    %> .. code-block:: matlab
    %>
    %>   ref.plot();
    %>   ref.plot( npts );
    %>
    %>   fmt1 = {'Color','blue','Linewidth',2}; % first arc of the biarc
    %>   fmt2 = {'Color','red','Linewidth',2};  % second arc of the biarc
    %>   ref.plot( npts, fmt1, fmt2 );
    %>
    %> \endrst
    %>
    %> - `npts`: number of sampling points for plotting
    %> - `fmt1`: format of the odd clothoids
    %> - `fmt2`: format of the even clothoids
    %>
    function plot( self, varargin )
      if nargin > 1
        npts = varargin{1};
      else
        npts = 400;
      end
      if nargin > 2
        fmt1 = varargin{2};
      else
        fmt1 = {'Color','red','LineWidth',3};
      end
      if nargin > 3
        fmt2 = varargin{3};
      else
        fmt2 = {'Color','blue','LineWidth',3};
      end
      for k=1:self.numSegments()
        C = self.get(k);
        if mod(k,2) == 0
          C.plot( npts, fmt1{:} );
        else
          C.plot( npts, fmt2{:} );
        end
        hold on;
      end
    end
    % - - - - - - - - - - - - - - - - - - - - - - - - - - - - - - - - - - -
    %> Plot the clothoid list with offset
    %>
    %> **Usage:**
    %>
    %> \rst
    %> .. code-block:: matlab
    %>
    %>   ref.plot_offs( offs, npts );
    %>
    %>   fmt1 = {'Color','blue','Linewidth',2}; % first arc of the biarc
    %>   fmt2 = {'Color','red','Linewidth',2};  % second arc of the biarc
    %>   ref.plot_offs( offs, npts, fmt1, fmt2 );
    %>
    %> \endrst
    %>
    %> - `npts`: number of sampling points for plotting
    %> - `fmt1`: format of the first arc
    %> - `fmt2`: format of the second arc
    %> - `offs`: offset used in the plotting
    %>
    function plot_offs( self, offs, npts, varargin )
      if nargin > 3
        fmt1 = varargin{1};
      else
        fmt1 = {'Color','red','LineWidth',3};
      end
      if nargin > 4
        fmt2 = varargin{2};
      else
        fmt2 = {'Color','blue','LineWidth',3};
      end
      for k=1:self.numSegments()
        C = self.get(k);
        if mod(k,2) == 0
          C.plot_offs( offs, npts, fmt1{:} );
        else
          C.plot_offs( offs, npts, fmt2{:} );
        end
        hold on;
      end
    end
    % - - - - - - - - - - - - - - - - - - - - - - - - - - - - - - - - - - -
    %> Plot the curvature of the clothoid list
    %>
    %> **Usage:**
    %>
    %> \rst
    %> .. code-block:: matlab
    %>
    %>   ref.plotCurvature( npts );
    %>
    %>   fmt1 = {'Color','blue','Linewidth',2};
    %>   fmt2 = {'Color','red','Linewidth',2};
    %>   ref.plotCurvature( npts, fmt1, fmt2 );
    %>
    %> \endrst
    %>
    %> - `npts`: number of sampling points for plotting
    %> - `fmt1`: format of the first arc
    %> - `fmt2`: format of the second arc
    %>
    function plotCurvature( self, npts, varargin )
      if nargin > 2
        fmt1 = varargin{1};
      else
        fmt1 = {'Color','red','LineWidth',3};
      end
      if nargin > 3
        fmt2 = varargin{2};
      else
        fmt2 = {'Color','blue','LineWidth',3};
      end
      s0 = 0;
      for k=1:self.numSegments()
        C  = self.get(k);
        ss = 0:C.length()/npts:C.length();
        [~,~,~,kappa] = C.evaluate(ss);
        if mod(k,2) == 0
          plot( s0+ss, kappa, fmt1{:} );
        else
          plot( s0+ss, kappa, fmt2{:} );
        end
        s0 = s0+ss(end);
        hold on;
      end
    end
    % - - - - - - - - - - - - - - - - - - - - - - - - - - - - - - - - - - -
    %> Plot the angle of the clothoid list
    %>
    %> **Usage:**
    %>
    %> \rst
    %> .. code-block:: matlab
    %>
    %>   ref.plotAngle( npts );
    %>
    %>   fmt1 = {'Color','blue','Linewidth',2};
    %>   fmt2 = {'Color','red','Linewidth',2};
    %>   ref.plotAngle( npts, fmt1, fmt2 );
    %>
    %> \endrst
    %>
    %> - `npts`: number of sampling points for plotting
    %> - `fmt1`: format of the first arc
    %> - `fmt2`: format of the second arc
    %>
    function plotAngle( self, npts, varargin )
      if nargin > 2
        fmt1 = varargin{1};
      else
        fmt1 = {'Color','red','LineWidth',3};
      end
      if nargin > 3
        fmt2 = varargin{2};
      else
        fmt2 = {'Color','blue','LineWidth',3};
      end
      s0 = 0;
      for k=1:self.numSegments()
        C  = self.get(k);
        ss = 0:C.length()/npts:C.length();
        [~,~,theta,~] = C.evaluate(ss);
        if mod(k,2) == 0
          plot( s0+ss, theta, fmt1{:} );
        else
          plot( s0+ss, theta, fmt2{:} );
        end
        s0 = s0+ss(end);
        hold on;
      end
    end
    % - - - - - - - - - - - - - - - - - - - - - - - - - - - - - - - - - - -
    %> Plot the normal of the clothoid list
    %>
    %> **Usage:**
    %>
    %> \rst
    %> .. code-block:: matlab
    %>
    %>   ref.plotNormal( step, len );
    %>
    %> \endrst
    %>
    %> - `step`: number of sampling normals
    %> - `len`:  length of the plotted normal
    %>
    function plotNormal( self, step, len )
      for k=1:self.numSegments()
        C = self.get(k);
        C.plotNormal( step, len );
      end
    end
    % - - - - - - - - - - - - - - - - - - - - - - - - - - - - - - - - - - -
    %> Save the clothoid list sampled on a file
    %>
    %> **Usage:**
    %>
    %> \rst
    %> .. code-block:: matlab
    %>
    %>   ref.saveSampled( filename, ds );
    %>
    %> \endrst
    %>
    %> - `filename`: file name
    %> - `ds`:       sample point every `ds`
    %>
    %> the file is of the form
    %> \rst
    %> .. code-block:: text
    %>
    %>   X Y THETA
    %>   0 0 1.2
    %>   ...
    %>   ...
    %>
    %> \endrst
    %>
    function saveSampled( self, filename, ds )
      fd = fopen( filename, 'w' );
      L  = self.length();
      n  = ceil( L / ds );
      fprintf(fd,'X\tY\tTHETA\n');
      for k=1:n
        s = (k-1)*L/(n-1);
        [x,y,theta] = self.evaluate( s );
        fprintf(fd,'%20.10g\t%20.10g\t%20.10g\n',x,y,theta);
      end
      fclose(fd);
    end
    % - - - - - - - - - - - - - - - - - - - - - - - - - - - - - - - - - - -
    %> Save the clothoid list on a file as a list of segments
    %>
    %> **Usage:**
    %>
    %> \rst
    %> .. code-block:: matlab
    %>
    %>   ref.saveClothoids( filename, ds );
    %>
    %> \endrst
    %>
    %> - `filename`: file name
    %> - `ds`:       sample point every `ds`
    %>
    %> the file is of the form
    %> \rst
    %> .. code-block:: text
    %>
    %>   x0 y0 theta0 kappa0 dk  L
    %>   0  0  1.2    0.0    0.1 2
    %>   ...
    %>   ...
    %>
    %> \endrst
    %>
    function saveClothoids( self, filename )
      fd = fopen( filename, 'w' );
      fprintf(fd,'x0\ty0\ttheta0\tkappa0\tdk\tL\n');
      for k=1:self.numSegments()
        C = self.get(k);
        [x0,y0,theta0,k0,dk,L] = C.getPars();
        fprintf(fd,'%20.10g\t%20.10g\t%20.10g\t%20.10g\t%20.10g\t%20.10g\n',x0,y0,theta0,k0,dk,L);
      end
      fclose(fd);
    end

  end

end
```

### Quick search

### Table of Contents

- Matlab Interface Manual
- C++ API
- MATLAB API

«
hide menu

menu
sidebar
»

### Navigation

- index
- toc
- Clothoids »
- Program Listing for File ClothoidList.m

© Copyright 2021, Enrico Bertolazzi and Marco Frego.
Created using Sphinx 4.2.0.
